# Comparison of Ultrastructure, Extracellular Matrix, and Drug Susceptibility in *M. avium* subs. *hominissuis* Biofilms

**DOI:** 10.3390/pathogens12121427

**Published:** 2023-12-08

**Authors:** William R. McManus, Jeffrey S. Schorey

**Affiliations:** Department of Biological Sciences, Galvin Life Science Center, University of Notre Dame, Notre Dame, IN 46556, USA; wmcmanus@nd.edu

**Keywords:** *Mycobacterium avium*, biofilm, resistance, extracellular matrix

## Abstract

Pulmonary infections with *Mycobacterium avium* occur in susceptible individuals following exposure to the bacterium in the environment, where it often persists in biofilms. Many methods have been used to generate biofilms of *M. avium*, and it is unknown whether different approaches generate similar structures and cell phenotypes. To make a parallel comparison of in vitro biofilm ultrastructure, extracellular matrix (ECM) composition, and the drug susceptibility of biofilm resident bacteria, we used two published methods to generate *M. avium* biofilms: four-week incubation in M63 medium or 24 h exposure to dithiothreitol (DTT). Scanning electron microscopy revealed differences in the biofilm ultrastructure between the two methods, including variation in the appearance of ECM materials and morphology of resident cells, while light microscopy and staining with calcofluor white indicated that both biofilms contained polysaccharides characteristic of cellulose. Measuring the susceptibility of biofilms to degradation by enzymes suggested differences in structurally important ECM molecules, with DTT biofilms having important protein and, to a lesser extent, cellulose components, and M63 biofilms having moderate protein, cellulose, and DNA components. Both biofilms conferred resistance to the bactericidal effects of amikacin and clarithromycin, with resident cells being killed at greater than 10-fold lower rates than planktonic cells at almost all concentrations. These comparisons indicate differences in biofilm responses by *M. avium* under differing conditions, but also suggest common features of biofilm formation, including cellulose production and antimicrobial resistance.

## 1. Introduction

*Mycobacterium avium* subs. *hominissuis* is an opportunistic pathogen, causing pulmonary infections in people whose respiratory systems have been damaged due to injuries or diseases such as cystic fibrosis, bronchiectasis, chronic obstructive pulmonary disease, or emphysema; people who are immunocompromised; and, increasingly, in elderly people without predisposing conditions, especially elderly women with slender physiques [1]. Infections with *M. avium* occur worldwide, and increasing incidences have been reported in resource-rich countries where longitudinal epidemiological studies have been possible [2,3,4]. Like many other species of nontuberculous mycobacteria (NTM), *M. avium* is commonly found in numerous natural and engineered habitats, including marginal niches where few species are able to survive due to low nutrient availability, low oxygen levels, and/or the presence of potentially toxic agents like chlorine or other disinfectants [5]. These include household reservoirs, such as showerheads, hot tubs, and humidifiers, that place people in frequent contact with *M. avium* and which have been shown to provide a route for infection via the inhalation of aerosols [6,7,8,9,10]. *M. avium* is able to persist in plumbing systems and other habitats by biofilm formation, a process in which exceptionally hydrophobic mycobacterial cells preferentially adhere to a surface or to particles in suspension, then undergo sessile growth and secrete an extracellular matrix (ECM) [11]. Growth of *M. avium* in biofilms has been observed to cause increased resistance of the bacterium to water treatment applications [12], disinfectants [13,14], and antimicrobial drugs [15,16]. These resistance phenotypes have led to the hypotheses that biofilm growth might condition *M. avium* to succeed as an opportunistic pathogen and that biofilm growth in vivo might explain the recalcitrance of *M. avium* pulmonary infections to therapy [17].

A limitation to our understanding of *M. avium* biofilms is the fact that studies have employed a variety of methods to cause biofilm formation in vitro, and these methods vary in many aspects, including temperature, nutrient and ion availability, substrate material for biofilm attachment, the presence or absence of chemical stressors, pH, and the presence or absence of flow. Most observations of *M. avium* biofilms have been made using only one method, and this raises uncertainty about whether observations made using one method are characteristic of *M. avium* biofilms broadly or whether they might be caused by conditions specific to that method. A side-by-side comparison of biofilms generated by multiple methods will help delineate which biofilm phenotypes are characteristic of *M. avium* biofilm formation mechanisms generally and which phenotypes appear in response to specific conditions.

To enable a parallel comparison of *M. avium* biofilms formed in vitro, we selected two previously published methods for producing surface-attached biofilms which varied in the media composition and time required for biofilm maturation. In the first method, cells of *M. avium* were incubated in a previously described M63-based biofilm medium for 4 weeks to allow for biofilm development over an extended period [18,19,20]. This extended time period is common in many models, and may reflect the gradual process of biofilm formation that likely occurs in the environment [11]. The other method used dithiothreitol (DTT)-induced thiol reductive stress to trigger rapid biofilm development, with biofilm formation occurring in one day [16,21,22]. The mechanism of this model may represent a biofilm formation response that could occur in vivo, where *M. avium* could experience intracellular thiol reductive stress from a host factor like glutathione [22,23]. In this study, we present a comparison of the ultrastructure appearance, biofilm ECM composition, and drug susceptibility of biofilm resident cells of *M. avium* grown in these two biofilm models. In this comparison, we observed differences in the appearance and composition of biofilms formed under the different methods, suggesting that *M. avium* employs different biofilm formation mechanisms in response to different stimuli. However, we also observed similarities, including polysaccharides characteristic of cellulose in the ECM and resistance of cells in biofilms to killing by antimicrobial drugs, indicating possible mechanisms and phenotypes common to *M. avium* biofilms in diverse conditions.

## 2. Materials and Methods

### 2.1. Bacterial Strains and Media

*Mycobacterium avium* subs. *hominissuis* strain A5 was originally isolated from the blood of a patient with AIDS with disseminated mycobacterial infection and strain 2151 was originally isolated from the sputum of a patient with pulmonary mycobacterial infection. These strains express different serotype-specific glycopeptidolipids (GPLs): A5 expresses serotype 4 and 2151 expresses serotype 2. The strains were cultured on Middlebrooks 7H10 agar (BD, Franklin Lakes, NJ, USA) containing 10% oleic acid-albumin-dextrose-catalase (OADC; BD, Franklin Lakes, NJ, USA) supplement at 37 °C and in Middlebrooks 7H9 broth (BD, Franklin Lakes, NJ, USA) containing 10% OADC at 37 °C with constant agitation. All experiments with *M. avium* were carried out at biosafety level 2.

### 2.2. Biofilm Culture Conditions

Both biofilm methods studied were adapted for biofilm growth on multiwell polystyrene plates (unless otherwise noted) at 37 °C and in stationary conditions. Growth of biofilms in M63 medium was based on previous studies [18,19]. *M. avium* strains were grown in liquid culture to an optical density 600 nm (OD600) of approximately 0.8, then pelleted and resuspended in biofilm medium to an OD600 = 0.2. The biofilm medium consisted of M63 medium (United States Biological, Salem, MA, USA) supplemented with 2% glucose, 0.5% casamino acids, 1 mM MgSO_4_, and 0.7 mM CaCl_2_. An appropriate volume (1 mL in a 24-well plate well, or equivalent scaled to the surface area of the bottom of other vessels) was dispensed into polystyrene plates, and plates were sealed with parafilm and incubated at 37 °C for four weeks. Phosphate-buffered saline (PBS) was added to interwell spaces to prevent evaporation. Biofilm formation using dithiothreitol (DTT) was based on previous studies [16,21]. *M. avium* strains were grown in liquid culture to an OD600 of approximately 0.8, then pelleted and resuspended in 7H9 broth + 5% OADC to an OD600 = 0.8. DTT (Roche, Mannheim, Germany) was added to a final concentration of 6 mM. An appropriate volume (1 mL in a 24-well plate well, or equivalent) was dispensed into polystyrene plates, and plates were incubated at 37 °C for 24 h.

### 2.3. Scanning Electron Microscopy (SEM)

Biofilms were grown as described above, with the addition of circular glass coverslips to the bottom of the multiwell plate wells. Following biofilm formation, the biofilm media supernatants were removed and the biofilms were rinsed with water. Coverslips with adherent biofilms were removed from the multiwell plates, and the samples (planktonic and biofilm) were dried overnight. Planktonic samples were prepared by resuspending *M. avium* grown in liquid culture in PBS, then allowing the bacterial suspension to dry on circular coverslips overnight. Then, the samples were fixed with 2% glutaraldehyde in 0.1 M sodium cacodylate (pH 7.5) for 1 h. Samples were rinsed with 0.1 M sodium cacodylate (pH 7.5), fixed in 1% osmium tetroxide, then rinsed again three times with buffer. The samples were then dehydrated using a graded ethanol series, before drying with a critical-point dryer. The dried samples were mounted on SEM stubs and sputter coated with iridium to a thickness of 5 µm. Samples were imaged at the Notre Dame Integrated Imaging Facility using a Magellan 400 XHR scanning electron microscope (FEI, Hillsboro, OR, USA).

### 2.4. Calcofluor White Staining

Biofilms were grown as described above, with volumes adjusted for 4-well chamber slides (900 µL biofilm culture per well). Bacteria grown in liquid culture and resuspended in PBS were added to 4-well chamber slides and allowed to dry in the wells overnight. Biofilm media supernatants were removed and the biofilms were rinsed with PBS. All samples were fixed in 10% formalin for 1 h, then rinsed three times with PBS, followed by staining with calcofluor white (3 µg/mL; Sigma Aldrich, Burlington, MA, USA) for 30 min. Samples were rinsed again, stained with Auramine M (BD) for 15 min, then destained with acid alcohol and rinsed with water. Finally, samples were mounted using mounting medium (Thermo Fisher, Waltham, MA, USA) and imaged using an inverted fluorescent microscope (Zeiss; Oberkochen, Germany). Auramine M was imaged using an excitation wavelength of 470 nm, and calcofluor white was imaged using an excitation wavelength of 365 nm.

### 2.5. Biofilm Enzymatic Degradation Assay

Biofilms were grown in 24-well polystyrene dishes, as described above. Biofilm media supernatants were removed and the biofilms were rinsed with PBS. Then, enzyme reaction buffers with or without enzymes were added to biofilm-containing wells in triplicate, and the biofilms were incubated at 37 °C for 6 h. Enzymes and buffers were formulated as follows: 0.1 mg/mL proteinase K (VWR, Radnor, PA, USA) in 30 mM Tris-HCl (pH 8.0), 1 mg/mL Cellulysin Cellulase (MilliporeSigma, Billerica, MA, USA) in 50 mM citrate buffer (pH 4.0), and 10 U/mL DNase I (Roche, Mannheim, Germany) in 200 mM Tris-HCl (pH 8.4), 20 mM MgCl_2_, 500 mM KCl. Following incubation, the enzyme or buffer supernatants were removed from the biofilms, and the biofilms were stained with 0.1% crystal violet for 10 min, rinsed twice with water, then allowed to dry overnight. To quantify the residual biomass, the crystal violet was solubilized in 30% acetic acid for 10 min with agitation and quantified by measuring the OD600 of the solution. To compare the average loss of biofilm biomass across 3–4 biological replicates, we used a one sample t-test comparing staining in enzyme-treated biofilms to respective buffer-treated controls.

### 2.6. Antimicrobial Susceptibility

Biofilms were grown as described above, with volumes adjusted for 48-well polystyrene plates (500 µL biofilm culture per well). Planktonic cultures were inoculated in normal growth medium at an OD600 = 0.1. Amikacin (Alfa Aesar, Haverhill, MA, USA) or clarithromycin (Sigma Aldrich, Burlington, MA, USA) dilutions were prepared in the growth medium corresponding to the different growth conditions (7H9 or M63), applied to the biofilm or planktonic bacteria, then incubated for 4 days at 37 °C. Then, the culture supernatants were removed, samples were resuspended in PBS, and biofilms were disrupted by scraping and pipetting in PBS + 0.5% Tween 80, and a 30 min incubation in a sonic bath (Thermo Fisher, Waltham, MA, USA). Serial dilutions of bacteria were made in PBS and plated on 7H10 agar + 10% OADC to quantify the surviving colony forming units (CFUs). Colonies were counted after 10 days of incubation at 37 °C. Plates were viewed at extended timepoints, but no additional colonies were observed.

## 3. Results

### 3.1. Biofilm Models Display Differences in Ultrastructure and Cell Morphologies

**Figure 1:**
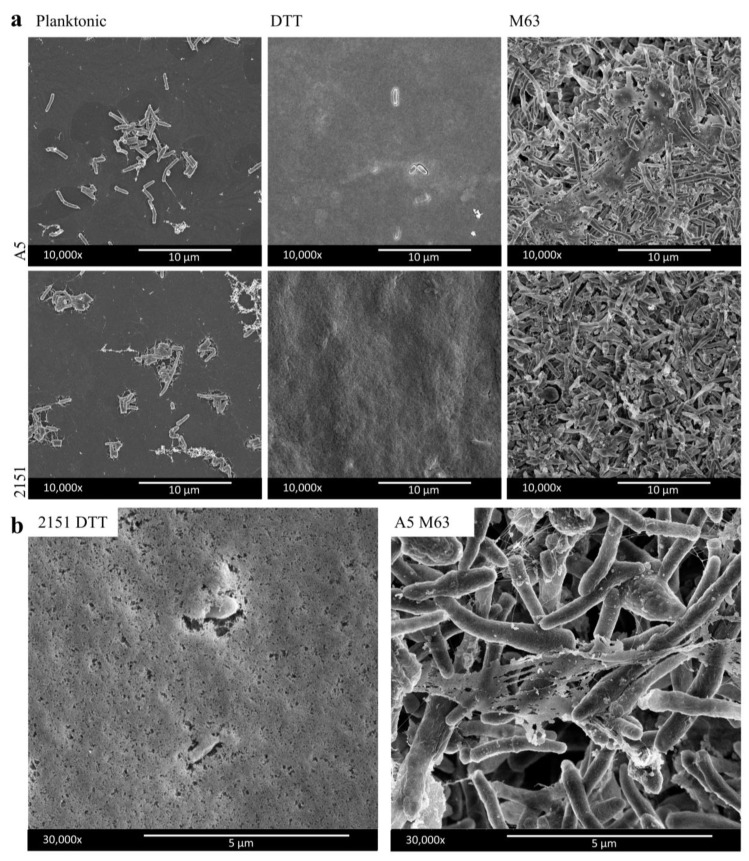
Ultrastructure appearance differs between DTT and M63 *M. avium* biofilm models. SEM was carried out on *M. avium* planktonic or biofilm samples grown on glass coverslips. (**a**) Representative images for each biofilm and planktonic condition for each *M. avium* strain at 10,000× magnification. (**b**) A 30,000× magnification showing ECM detail of select representative images from each biofilm model.

The general ultrastructure appearances of biofilms formed by each in vitro method were observed using scanning electron microscopy (SEM). Biofilms and planktonic samples of two strains of *M. avium* (A5 and 2151) were included in each comparison to observe strain-specific effects. The strains in this study represent different serovariants, each expressing a distinct serotype-specific glycopeptidolipid (GPL): A5 is a serotype 4 strain and 2151 is a serotype 2 strain. The different methods of in vitro biofilm formation resulted in biofilms with distinct ultrastructure appearances that were similar between strains within a given method (Figure 1). The induction of biofilm formation with DTT gave rise to biofilms with relatively homogenous appearances, with few bacteria visible under a blanket of extracellular material (Figure 1). While few bacteria could be seen in most areas of the samples, this did not indicate an absence of bacteria, as imaging of a sample where the biofilm was disrupted during preparation, as well as some areas of the intact samples, revealed abundant bacilli beneath the homogenous material (Figure S1). In contrast, biofilm formation in M63 medium resulted in biofilms with heterogeneous extracellular material appearing as sheets, threads, and granules and many visible cells with heterogeneous morphologies, including elongation and branching (Figure 1).

### 3.2. Calcofluor White Staining Suggests the Presence of Cellulose in Biofilm ECM in Both Models

**Figure 2:**
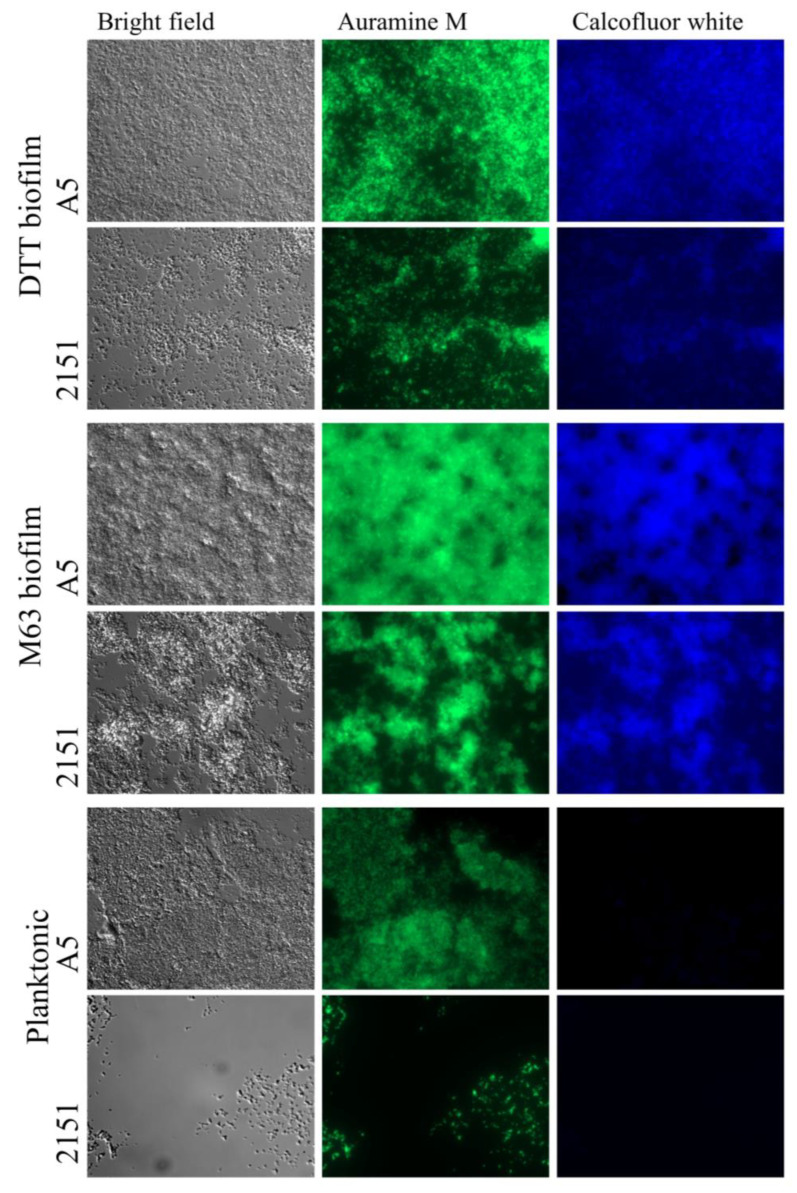
DTT and M63 biofilms both contain cellulose-like polysaccharides. Biofilm or planktonic specimens of two *M. avium* strains (A5 and 2151) were grown and fixed in chamber slides, then stained with calcofluor white (3 μg/mL) to detect the β(1→4) linked D-glucose polysaccharides. Samples were then rinsed and stained with Auramine M for detection of the mycobacterial cell envelope, then destained with acid alcohol and rinsed with water. Representative images are shown, visualized at 1000× magnification on a Zeiss inverted fluorescence microscope.

Previous studies characterizing the DTT-induced biofilm model [16] demonstrated the presence of cellulose in that model with multiple mycobacterial species, including *M. avium*. Cellulose was also identified by one study in the ECM of *M. smegmatis* biofilms formed in M63 medium [20]. To compare the presence of polysaccharides with β(1→4) linked D-glucose units (characteristic of cellulose) in specimens from both biofilm models and planktonic culture, samples were stained with calcofluor white and counterstained with Auramine M to visualize the mycobacterial cell wall. Relative to the planktonic bacteria controls, where no calcofluor white staining was detected, increased staining was observed in both biofilm models for each strain (Figure 2).

### 3.3. Biofilm Models Differ in Structurally Important ECM Molecules

**Figure 3:**
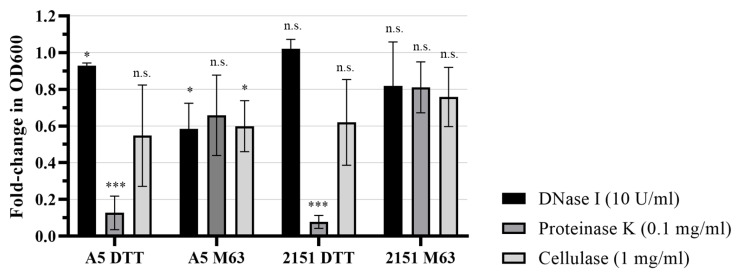
M63 and DTT biofilms have differing susceptibilities to enzymatic degradation. Biofilms were treated with enzymes or enzyme reaction buffer for 6 h, then stained for 10 min with 0.1% crystal violet and rinsed twice with water. The stained biomass was dried overnight, then the crystal violet was solubilized in 30% acetic acid and the OD600 was quantified. Fold-change in biofilm biomass was calculated as the ratio of enzyme-treated biofilm to paired buffer-treated biofilm crystal violet staining, and the plot shows the average and standard deviation of 3–4 biological replicates. Statistics: one sample *t*-test comparing enzyme-treated biofilms to respective buffer-treated controls: * *p* < 0.05, *** *p* < 0.001.

To further compare the characteristics of the ECM in each biofilm model, we sought to infer structurally important ECM molecules by exposing biofilms to DNase I, Proteinase K, and Cellulase to cause targeted degradation of ECM molecules that have been identified in previous studies [16,24]. In each biofilm model, the two *M. avium* strains assayed displayed similar patterns of susceptibility to degradation by the different enzymes, suggesting that the structurally important ECM molecules are similar between strains within a given model (Figure 3). The biofilms generated using the DTT model were significantly degraded by Proteinase K, resulting in 87.3% and 92.2% reductions in biomass in strains A5 and 2151, respectively. Degradation by Cellulase was moderate, though variable between replicates, resulting in 45% and 38% reductions in biomass in strains A5 and 2151, respectively. DNase caused no degradation in DTT biofilms of strain 2151 biofilms, and a slight (7%) reduction in biomass in DTT biofilms of strain A5 (Figure 3). Biofilms grown in M63 biofilm medium were moderately susceptible to degradation by each enzyme tested, suggesting heterogenous ECM composition, and biofilms of strain A5 exhibited higher degradation by each enzyme than biofilms of strain 2151. For strain A5 and 2151 biofilms, DNase caused biomass reductions of 42% and 18%, Proteinase K caused biomass reductions of 34% and 19%, and Cellulase caused biomass reductions of 40% and 24%, respectively (Figure 3).

### 3.4. Biofilm Resident M. avium Is Resistant to Killing by Antimicrobial Drugs

**Figure 4:**
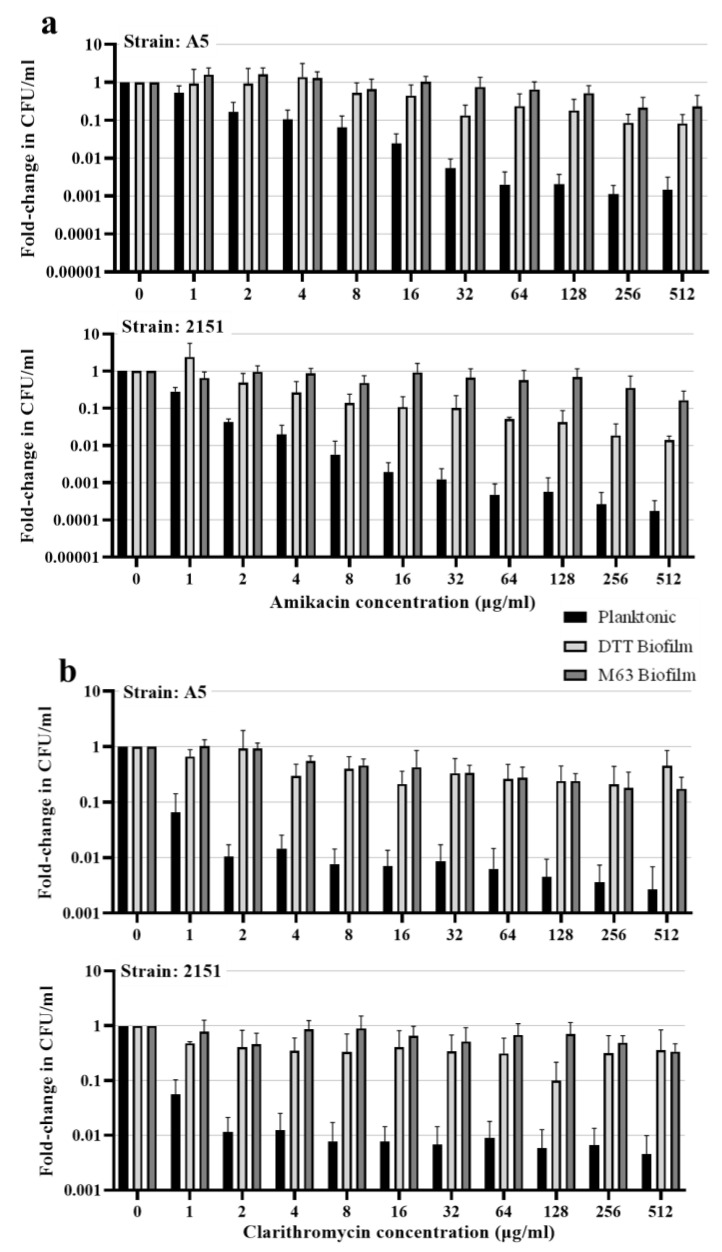
Growth of *M. avium* in biofilms confers resistance to antimycobacterial drugs. *M. avium* strains A5 and 2151 grown in planktonic culture, DTT biofilm, or M63 biofilm were exposed to varying concentrations of (**a**) amikacin or (**b**) clarithromycin for 4 days. After 4 days, supernatants were removed and biofilms were disrupted by scraping, pipetting, and vortexing, and dilutions of each sample were plated on 7H10 agar + 10% OADC to quantify CFUs. For each condition, counts were normalized to the untreated (0 μg/mL) count. The plot shows the average fold-change and standard deviation for 3–4 biological replicates.

To assess resistance to killing by two drugs commonly prescribed to treat *M. avium* pulmonary disease, clarithromycin and amikacin, we exposed intact biofilms or planktonic cultures of both strains of *M. avium* to a range of concentrations of each drug for four days. In both strains, each biofilm model increased the resistance of bacteria to killing by either drug (Figure 4). Amikacin killed >90% of planktonic *M. avium* A5 at 8 μg/mL and >99% at 32 μg/mL. Planktonic *M. avium* 2151 was more sensitive to killing by amikacin, with >90% killed at 2 μg/mL, >99% killed at 8 μg/mL, and >99.9% killed at 64 μg/mL. In contrast, when bacteria were grown in DTT biofilms, both strains required 32× higher concentrations of amikacin (256 μg/mL in A5 and 64 μg/mL in 2151) for >90% killing, and in neither strain were >99% of bacteria killed even at the highest drug concentration tested (512 μg/mL) (Figure 4a). Resistance to killing was even higher in M63 biofilms, where the highest concentration of amikacin (512 μg/mL) did not kill >90% of *M. avium* of either strain in M63 biofilms (Figure 4a). Resistance to clarithromycin in both biofilm models followed a similar pattern. In both strains, clarithromycin killed >90% of planktonic bacteria at 1 μg/mL and >99% at 8 μg/mL (Figure 4b). However, in both biofilm models, the drug failed to kill 90% of bacteria of either strain at the highest concentrations tested. Plots showing the raw CFU values are included in Supplementary Figure S2, showing similar baseline levels of viable *M. avium* in each condition.

## 4. Discussion

In this study, we compared two methods for growing *M. avium* biofilms in vitro, which cause biofilm formation by markedly different stimuli. The M63 biofilm medium, four-week incubation method likely models the gradual process of biofilm formation that occurs in the environment, in which individual cells adhere to a surface, undergo sessile growth, and secrete an ECM over an extended period of time [18]. Conversely, the DTT-induced, one-day incubation method models conditions the opportunistic pathogen might encounter in a potential host [16,21,22]. These include potential sources of thiol reductive stress, such as glutathione [23], and aggregated protein, which is present in the DTT-induced model in the form of albumin, the presence of which is necessary for biofilm formation to occur [22]. These two methods resulted in biofilms with apparent ultrastructure differences, as observed by SEM. Similar to SEM observations of DTT-induced biofilms of *M. tuberculosis* [21], the DTT-induced biofilms of both strains of *M. avium* were characterized by a homogenous ECM that concealed the cells within the biofilm. In contrast, the M63-incubated biofilms had extracellular materials with a variety of appearances and many visible cells with variable phenotypes, including elongation and branching. This heterogenous ultrastructure appearance has similarities to previous imaging of *M. avium* biofilms grown with similarly extended incubation times [24,25]. The observation of abnormal bacterial cell morphologies raises the possibility that aberrations from the normal cell division processes may occur when *M. avium* resides in a biofilm for weeks or months. Previous studies in *M. smegmatis* observed that both the depletion and the overexpression of the tubulin-like protein FtsZ, which is responsible for septum formation in mycobacteria, results in cells with abnormal elongation and branching phenotypes, and this protein could serve as a starting point for further study into the unusual morphologies observed [26,27,28]. In this study, we found it necessary to include a pre-fixation air drying step to improve sample retention on glass coverslips during the fixation process for SEM. In studies of other bacterial biofilms, air-drying has been used to improve sample retention on mineral substrates, though it has been observed to cause distortion of bacterial cells [29]. However, it is likely that the desiccation-resistant mycobacterial cell wall is less susceptible to these effects; this idea is supported by our observation of normal cell morphologies in our planktonic *M. avium* samples and in our biofilm samples where bacteria were visible (Figure 1). It is also possible that air drying and the standard SEM fixation process could cause the distortion of ECM structures in the biofilm, since the standard dehydrating process during fixation for SEM is known to cause some distortion of ECM structures [30], and air drying could have a similar effect. In spite of these limitations in our SEM method, we were able to observe differences in ECM appearance and cell morphology between the *M. avium* biofilm models studied and, for each method, were able to observe similarities between images of our samples and SEM images of samples from previous studies using the same or similar mycobacterial biofilm culture methods [21,24,25].

Consistent with the apparent differences in ultrastructure between models, the enzymatic degradation of biofilms with enzymes specific to ECM molecules identified in previous studies indicated differences in structurally important molecules between the models. In particular, the DTT biofilm model resulted in biofilms that were readily degraded by Proteinase K, in agreement with the finding of a previous study in *M. tuberculosis* [21], indicating a structurally essential protein component in the ECM of those biofilms, while M63 biofilms were degraded more moderately by Proteinase K. Since the presence of albumin in the culture medium is necessary for DTT-stimulated biofilm formation [22], it is possible that this is the major source of protein in the biofilm ECM in that model. The models also differed in susceptibility to DNase degradation: biofilms formed in M63 medium were moderately degraded by DNase (with strain A5 biofilms degraded to a larger extent that strain 2151 biofilms), while biofilms formed by DTT stimulation had little to no susceptibility to degradation by the enzyme, consistent with previous characterization of the DTT biofilm model in *M. tuberculosis* [21]. Previously, the contribution of extracellular DNA (eDNA) to the *M. avium* biofilm ECM was characterized in studies using a biofilm model based on a seven-day incubation of bacteria in Hank’s Balanced Salt Solution [24,31], where the authors observed strain-specific differences in the abundance of eDNA produced by *M. avium*, with strain A5 producing more eDNA than the other strains studied, in agreement with our observation that strain A5 M63 biofilms had greater susceptibility to DNase I degradation than strain 2151 M63 biofilms.

Notable similarities were also observed in our comparison of the biofilm ECM in the two models studied. Staining for β(1→4) linked D-glucose polysaccharides with calcofluor white suggested that both biofilm models produced biofilms with cellulose content in the ECM. Taken with the observation of moderate biofilm degradation by Cellulase in both models, these findings suggest that the production of cellulose may be a biofilm formation behavior employed by *M. avium* generally. In our study, we observed more modest degradation of the DTT biofilm by cellulase than a previous study [21], where DTT biofilms of *M. tuberculosis* were almost totally disrupted by the enzyme, possibly indicating species-specific differences between *M. avium* and *M. tuberculosis*. The production of cellulose by *M. avium* and mycobacteria more broadly was discovered only recently [21], and the genetic basis for this capability is unknown, as mycobacteria lack the classical cellulose synthase gene found in most bacteria [20,32]. In addition to cellulose in the mycobacterial biofilm ECM, *M. tuberculosis* has been observed to produce cellulose in the context of pulmonary infection [16]. Intriguingly, a recent study characterized a glycoside hydrolase family 6 (GH6) cellulase enzyme in *M. smegmatis* with high amino acid similarity to the *M. tuberculosis* cellulase CelA (88% similarity) and to an *M. avium* GH6 enzyme (82% similarity) [20]. In that study, the cellulase enzyme was not used by *M. smegmatis* for the metabolism of cellulose, as is the case in other cellulolytic bacteria, but was found to prevent biofilm formation when overexpressed and to disrupt the biofilm ECM of the bacterium when added exogenously. This suggests the possibility that this cellulase could have a functional role in biofilm modeling by reverting bacteria in a biofilm back into the planktonic state, similar to a behavior observed previously in the cellulolytic bacterium *Acetivibrio thermocellus* [20,33]. Our observation of cellulose in biofilms formed by *M. avium* in response to different stimuli adds further motivation to fully characterize the mechanism of cellulose production in mycobacteria, the stimuli that trigger cellulose production, the role of mycobacterial cellulase enzymes, and the relevance of these processes for both biofilm and in vivo persistence.

The biofilm models were also similar in their conferral of resistance to killing by clarithromycin and amikacin. Cells residing in either biofilm model were killed at >10-fold lower rates than planktonic cells at almost all concentrations tested. This is in general agreement with other studies of each model, where increased resistance has been observed previously: *M. avium* and other NTM in DTT-induced biofilms have been shown to be resistant to bedaquiline [16], and cells of *M. smegmatis* grown in M63 biofilms have been shown to have increased resistance to rifampin [20]. There are multiple potential explanations for the resistance observed in each model, and the differences in media and incubation time likely lead to some differences between models in the contributing mechanisms. However, it is possible that, despite the differences in the models, there may be common mechanisms of increased resistance in biofilm resident *M. avium*. Studies of *M. avium* grown in other models have demonstrated that bacteria grown in biofilms initially retain high levels of resistance to killing by antimicrobial drugs and disinfectants when resuspended, then lose resistance after 24 h of growth in liquid culture, suggesting that changes in gene expression play a role in resistance [14,34]. Therefore, future studies comparing the overall gene expression of bacteria residing in different biofilm models may offer leads toward discovering the adaptations in gene expression leading to resistance that are common to *M. avium* biofilms formed under differing conditions.

The design of this study, in which we compared aspects of *M. avium* biofilm appearance, ECM composition, and resident cell phenotypes between two biofilm models, should serve as a template for expanded parallel analyses that could include additional biofilm models and employ additional methods to characterize the biofilms and their constituent cells. Characterization and comparison of the involvement of membrane lipids in *M. avium* biofilms would be particularly informative, as studies of these molecules in the context of biofilms have been carried out almost entirely in other mycobacterial species. For instance, *M. tuberculosis* and *M. smegmatis* pellicle (liquid–air interface) biofilms contain short-chain mycolic acids in the ECM [35,36,37,38], and these have not been assayed in biofilms of other species. Additionally, *M. avium* and several other NTM express glycopeptidolipids (GPLs), which are necessary for biofilm formation [18,19,39], may have altered expression under biofilm conditions [40], and also appear to be immunologically important in the context of exposure and infection [41]. Further characterization of the GPL content and localization in *M. avium* biofilms is necessary to determine the specific role that these molecules play in biofilm formation.

## Data Availability

Data from this study can be found as part of the manuscript.

## Supplementary Materials

The following supporting information can be downloaded at: https://www.mdpi.com/article/10.3390/pathogens12121427/s1, Figure S1: Additional images of DTT-induced biofilms, showing visible bacteria cells; Figure S2: Antibiotic susceptibility assessment raw CFU plots.
