# Supplementary material for: Comparison of Ultrastructure, Extracellular Matrix, and Drug Susceptibility in M. avium subs. hominissuis Biofilms
Source: Pathogens. 2023 Dec 8;12(12):1427. doi: 10.3390/pathogens12121427 (PMC10747021; doi:10.3390/pathogens12121427)
Supplement: Supplementary file 1 [file pathogens-12-01427-s001.zip › pathogens-2695387-supplementary.pptx]

## Slide 1
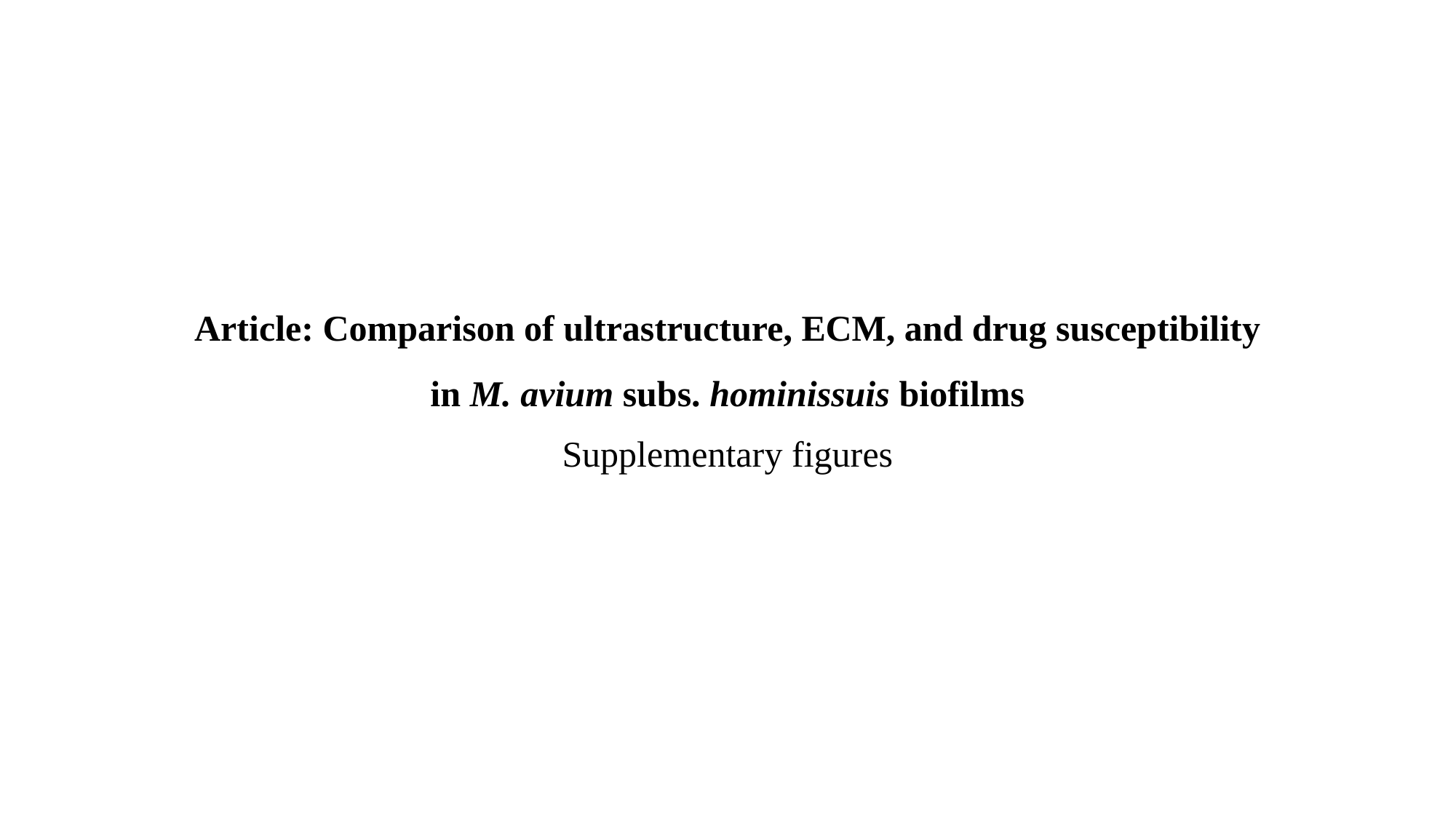

# Article: Comparison of ultrastructure, ECM, and drug susceptibility in M. avium subs. hominissuis biofilms
Supplementary figures

## Slide 2
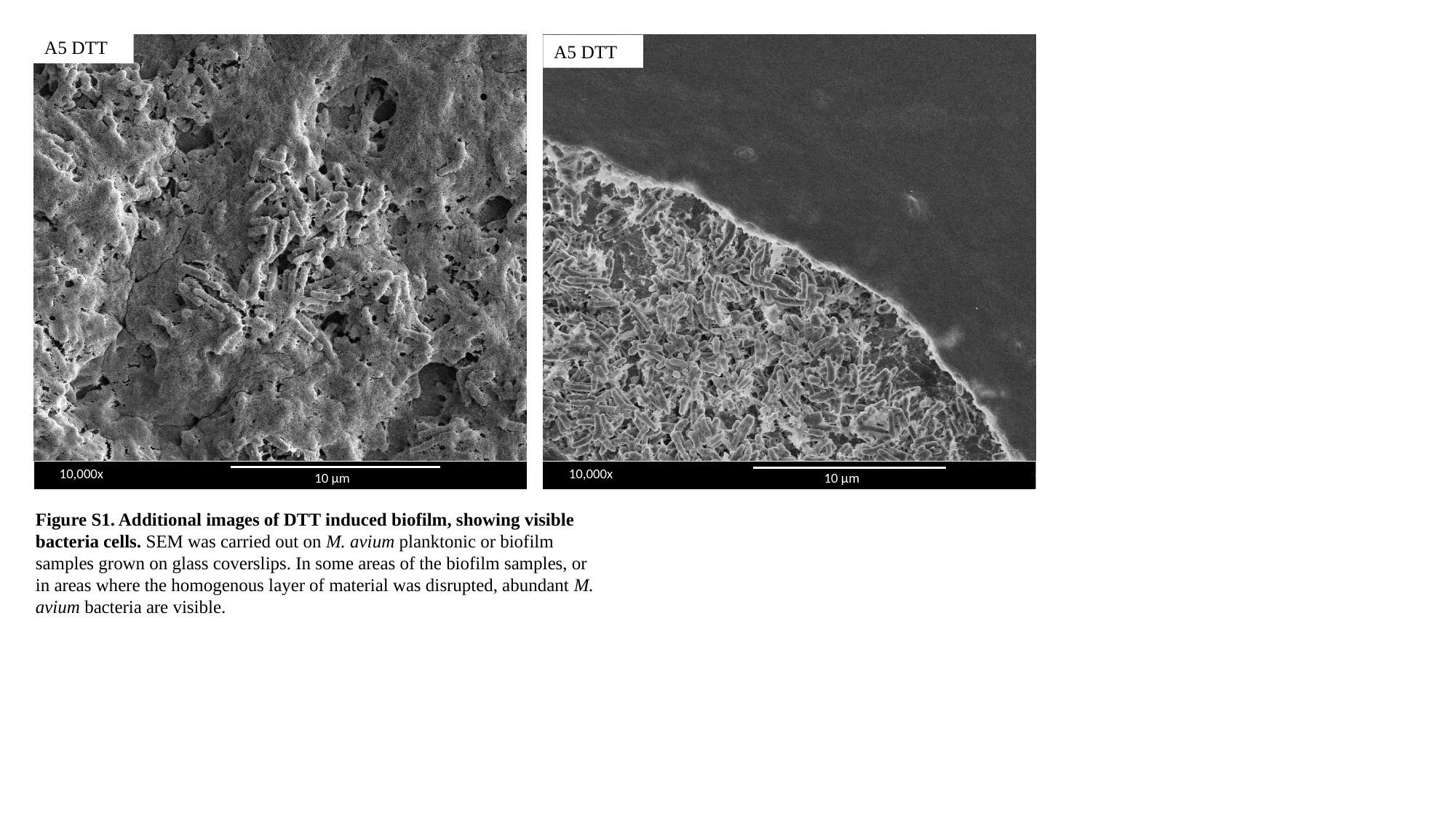

A5 DTT
A5 DTT
10,000x
10 μm
10,000x
10 μm
Figure S1. Additional images of DTT induced biofilm, showing visible bacteria cells. SEM was carried out on M. avium planktonic or biofilm samples grown on glass coverslips. In some areas of the biofilm samples, or in areas where the homogenous layer of material was disrupted, abundant M. avium bacteria are visible.

## Slide 3
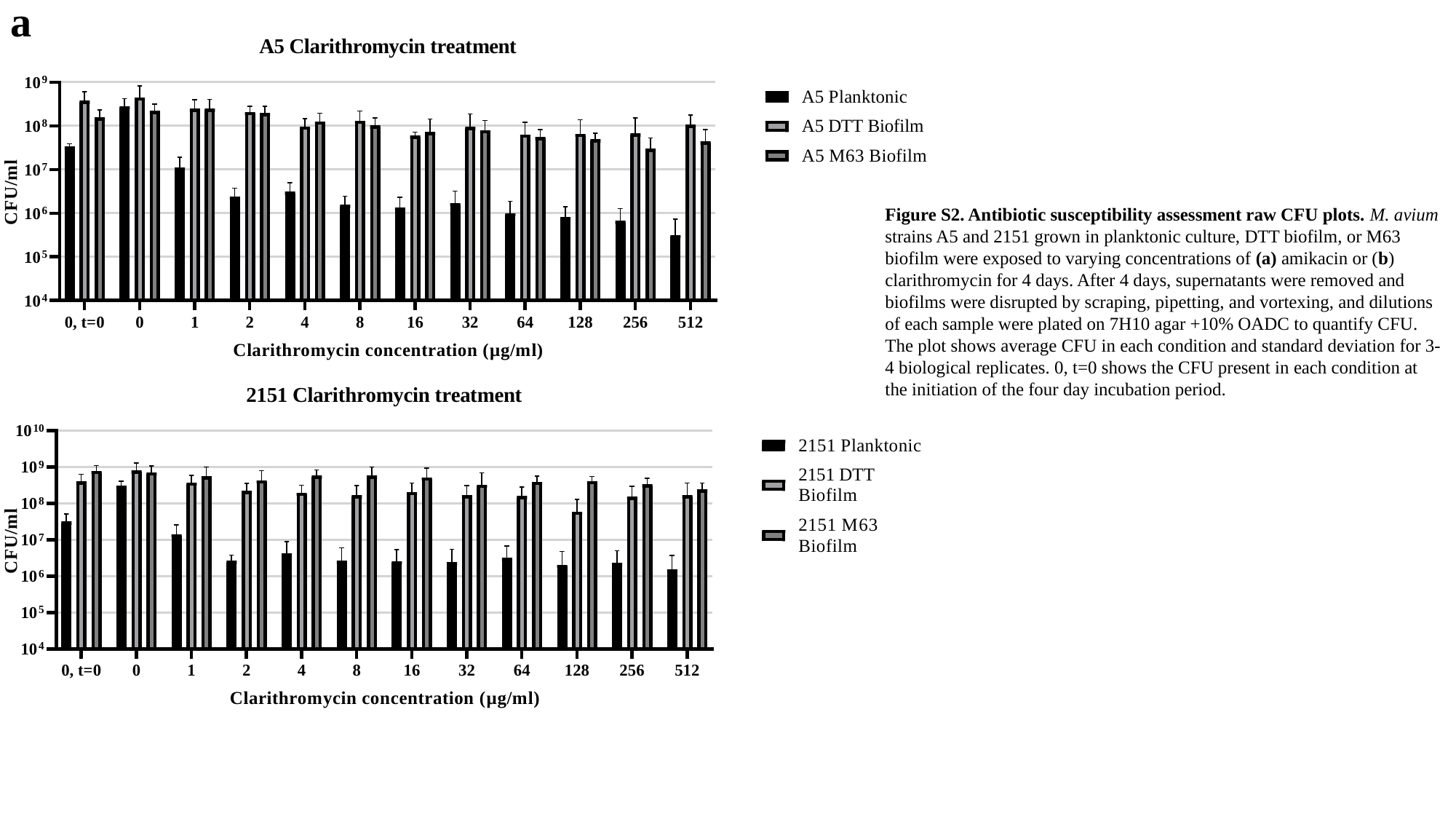

a
Figure S2. Antibiotic susceptibility assessment raw CFU plots. M. avium strains A5 and 2151 grown in planktonic culture, DTT biofilm, or M63 biofilm were exposed to varying concentrations of (a) amikacin or (b) clarithromycin for 4 days. After 4 days, supernatants were removed and biofilms were disrupted by scraping, pipetting, and vortexing, and dilutions of each sample were plated on 7H10 agar +10% OADC to quantify CFU. The plot shows average CFU in each condition and standard deviation for 3-4 biological replicates. 0, t=0 shows the CFU present in each condition at the initiation of the four day incubation period.

## Slide 4
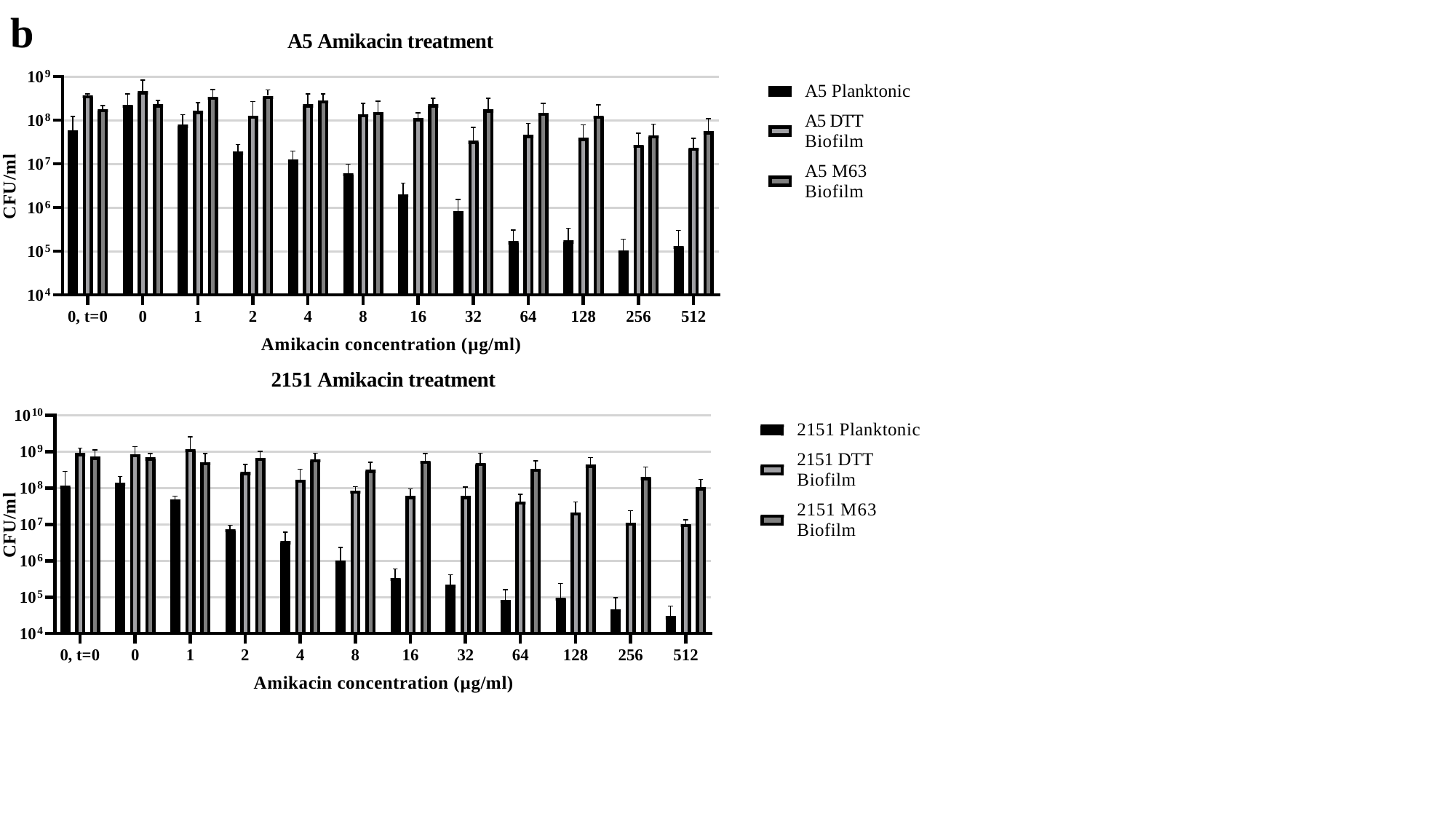

b
